# Supplementary material for: TREX2 component PCID2 scaffolds alternative SAC3-based subcomplexes with distinct RNA processing and export function
Source: bioRxiv. 2026 Apr 14:2026.04.13.716336. Preprint. [Version 1] doi: 10.64898/2026.04.13.716336 (PMC13105079; doi:10.64898/2026.04.13.716336)
Supplement: Supplement 1 [file NIHPP2026.04.13.716336v1-supplement-1.pdf]

bioRxiv preprint doi: https://doi.org/10.64898/2026.04.13.716336; this version posted April 14, 2026. The copyright holder for this preprint (which was not certified by peer review) is the author/funder. This article is a US Government work. It is not subject to copyright under 17 USC 105 and is also made available for use under a CC0 license.

Figure S1. TREX2 subunits differentially expressed across human tissues. GTEx heat map of TREX2 subunits and TPR RNA transcripts abundancy across 54 human tissues. The heat map is ordered by tissue clustering. A gradient from light yellow to dark blue represents abundance of transcripts across tissues.

Figure S2. TREX2 subunits, LENG8 and SAC3D1 gene targeting strategy. **a,** A scheme of endogenous targeting of TREX2 subunits and TREX2 subunits' associated protein partners with hygromycin-P2A-AID-NG/HA/FLAG tags. A position of two primer sets used for genotyping is shown relative to homologous arms. All genes were N-terminally targeted. F*out*-R*out* primer set anneals outside of the homologous arms, F*in*- R*in* primer set anneals inside of the homologous arms. **b**, A scheme of *Oryza sativa* TIR1 genomic integration into the *RCC1* gene locus. Two alternative types of TIR1 and auxin have been used in this study: a canonical OsTIR1 along with 1 mM Indole-3-acetic acid sodium salt and OsTIR1_F74G mutant along with 1 uM 5-Ph-IAA (42). **c**, A list of cell lines used in this study. **d**, Molecular weight of endogenous and HA- or NG/mCherry- tagged TREX2 subunits and TREX2 subunits' associated protein partners. Last column shows commercial antibody used to detect proteins on Western blot to confirm targeting and degradation. **e-i**, Genomic PCR of homozygous clones demonstrating the integration of the Hygromycin-P2A-AID-HA/FLAG sequence into genomic loci of ENY2 (e), CETN2 (f), CETN3 (g), LENG8 (h), and SAC3D1 (i) genes with *out-out* primer set. AID – Auxin Inducible Degron, HA - human influenza hemagglutinin. **j-l**, Genomic PCR of homozygous clones demonstrating the integration of the Hygromycin-P2A-NG sequence into genomic loci of PCID2 (j), ENY2 (k), and GANP (l) genes. NG – Neon Green fluorescent protein. NG-tagged cell lines (j-l) were used for IP-MS experiment, see Figure 3.

Figure S3. Binding partners of the TREX2 subunits under LS purification condition and *in silico* AF screen of the SAGA complex subunits. a, A silver gel of NG-GANP, NG-PCID2, NG-

bioRxiv preprint doi: https://doi.org/10.64898/2026.04.13.716336; this version posted April 14, 2026. The copyright holder for this preprint (which was not certified by peer review) is the author/funder. This article is a US Government work. It is not subject to copyright under 17 USC 105 and is also made available for use under a CC0 license.

ENY2, HA-CETN2, and FLAG-CETN3 and associated proteins isolated by the immunoprecipitation using the low salt buffer and NG-, HA, or FLAG-beads, respectively. The DLD-1 cell line was used as a control to assess background binding of the beads. NG-GANP, NG-PCID2, and NG-ENY2 are indicated with arrows. **b**, A schematic of SAGA complex domains. **c**, A quartile plot of ENY2 protein partners highlighting SAGA complex subunits immunoprecipitated with NG-ENY2. The right axis shows the protein coverage in the MS divided by its molecular weight. The left axis indicates the sum of the predicted Template Modeling score (pTM) and interface pTM score (ipTM), which are measures of predicted structure accuracy generated by AF for the protein and protein-protein binding interphase. The score 0 indicates no interaction between the bait and the prey; the score 1 indicates a high chance of both proteins interacting. DUB, HAT, SPL, Core, and TRRAP domain subunits are colored in violet, cerulean, light grey, yellow, dark grey colors, respectively on graph and schematic. **d**, *In silico* AF screen of known SAGA complex subunits interacting partners. Interacting surfaces were determined in Chimera X based on Cryo-EM PDB files. **e**, The visualization of interacting residues of selected SAGA subunits in Chimera X. The structures are derived from the 3D atomic structures of the cryo-EM density maps of 7KTS or 8H7G. The corresponding AF scores of the multimers are shown in the table, above or below each example. **f-h**, A quartile scatter plots of GANP (f), ENY2 (g), and PCID2 (h) binding partners under low salt (LS) purification condition. **i**, A Venn diagram representing the number of overlapping targets among binding partners of GANP, PCID2, ENY2, CETN2, or CETN3.

Figure S4. Identification of novel PCID2 interacting partners. A heat map comparing the MS abundance (in green) of PCID2 binding partners across the NG-GANP, NG-PCID2, and NG-ENY2 cell lines.

Figure S5. Identification of LENG8 and SAC3D1 interacting partners. a, A scheme of endogenous targeting of LENG8 and SAC3D1 with NG or AID-HA tags. **b**, An example of western blotting showing precipitation of HA-tagged LENG8 and SAC3D1 from DLD-1, AID-HA-LENG8, and AID-HA-SAC3D1 cell lines under high salt extraction conditions. **c-h**, Quartile plots showing LENG8 and SAC3D1 binding partners under high (HS) and low salt (LS) purification conditions. **i-k**, Predicted aligned error (PAE) plots of LENG8:PCID2, GANP:PCID2, and SAC3D1:PCID2 multimers.

bioRxiv preprint doi: https://doi.org/10.64898/2026.04.13.716336; this version posted April 14, 2026. The copyright holder for this preprint (which was not certified by peer review) is the author/funder. This article is a US Government work. It is not subject to copyright under 17 USC 105 and is also made available for use under a CC0 license.

Figure S6. Intracellular localization of endogenous and tagged LENG8 and SAC3D1. **a**, Live imaging of NG-tagged LENG8 and SAC3D1 in DLD-1 cell line. **b-c**, Intracellular localization of HA-tagged and untagged SAC3D1 (b) and LENG8 (c) using four alternative fixation condition methods. PFA – 4% Paraformaldehyde; PFA + 0.5% Triton – cells permeabilized simultaneously; nuclear-enriched – cells washed with buffer A (20 mM HEPES, pH 7.8; 2 mM DTT, 10% sucrose, 5 mM $MgCl_2$, 5 mM EGTA, 1% TX100, 0.075% SDS) for 5 min and fixed with 4% PFA; MeOH – cells fixed and permeabilized with methanol. **d-e**, Western blotting of untreated or 5-Ph-IAA treated AID-HA-SAC3D1 (d) and AID-HA-LENG8 (e) cell lines. Note the degradation of SAC3D1 and LENG8 proteins happens within 1 h and 45 min, respectively. *Indicate replacement of culture media with fresh media containing 5-Ph-IAA every 30 min. **f**, A single plane image and estimation plots showing the difference between means in the nuclear-to-cytoplasmic (Nu/Cyt) localization ratio of mCherry-PCID2 in untreated cells compared to those with an acute loss of SAC3D1. Data are presented as mean values, ****p-value < 0.0001, unpaired Student's *t*-test. Two separate clones were analyzed: clone 5, n = 52 untreated and n = 59 5-Ph-IAA-treated cells; clone 6, n = 57 untreated and n = 53 5-Ph-IAA-treated cells.

Figure S7. LENG8-bound proteins enriched in nuclear speckles. a, STRING map of SAC3D1 interacting partners reproduced in at least two independent purification conditions with 5-fold enrichment. Circles corresponding to proteins with AF scores 0.6 and higher are enlarged respectively. **b**, A Venn diagram of LENG8 binding partners showing the overlap with spliceosome and spliceosome-associated proteins. **c**, A Venn diagram illustrating the association of LENG8-spliceosome-associated proteins (99 candidates from b) with the pool of nucleoplasmic proteins and proteins localized in nuclear speckles. **d-e**, Co-localization of nuclear speckle marker SC35 and top LENG8 interacting partners in untreated DLD-1 cells and upon LENG8 loss (**e**). **f**, AF multimers of PCID2 protein with LENG8 or SAC3D1, and DDX39B. **g**, A heat map of AF prediction scores for LENG8, PCID2, SAC3D1, GANP, ENY2 and spliceosome components.

Figure S8. Gene expression changes upon LENG8 loss. a, A proportion of RNA transcript subtypes in LENG8 RNA-seq data. Only significantly changed differentially expressed RNAs were taken into analysis with log2FC > 20%, adj. p-value < 0.05. **b-c**, Top GO-terms (Biological Process, **b**; Molecular Function, **c**) of differentially expressed RNAs upon LENG8 loss (HumanMine v12, 2022).

bioRxiv preprint doi: https://doi.org/10.64898/2026.04.13.716336; this version posted April 14, 2026. The copyright holder for this preprint (which was not certified by peer review) is the author/funder. This article is a US Government work. It is not subject to copyright under 17 USC 105 and is also made available for use under a CC0 license.

Figure S9. Examples of alternative polyadenylation site usage events upon LENG8 loss. a-f, IGV snapshots of RNA-Seq data, RT-qPCR analysis of upregulated regions (blue line), and agarose gel electrophoresis of the RT-qPCR products between the last exon and the joining upregulated intron (green line) for *rps28* (**a**), *psmd3* (**b**), *ing3* (**c**), *siva1* (**d**), and *znf789* (**e**) transcripts. Regions with alternative polyadenylation usage events with increased read counts are highlighted with dashed squares. The frequency of PAS usage and fold change of the upregulated region are indicated, respectively. RT-qPCR graphs represent the mean value of three technical replicates of one experiment; error bars are SD. Asterisks indicate ****p-value < 0.0001 and ***< 0.01 in unpaired two-tailed Student's *t*-test.

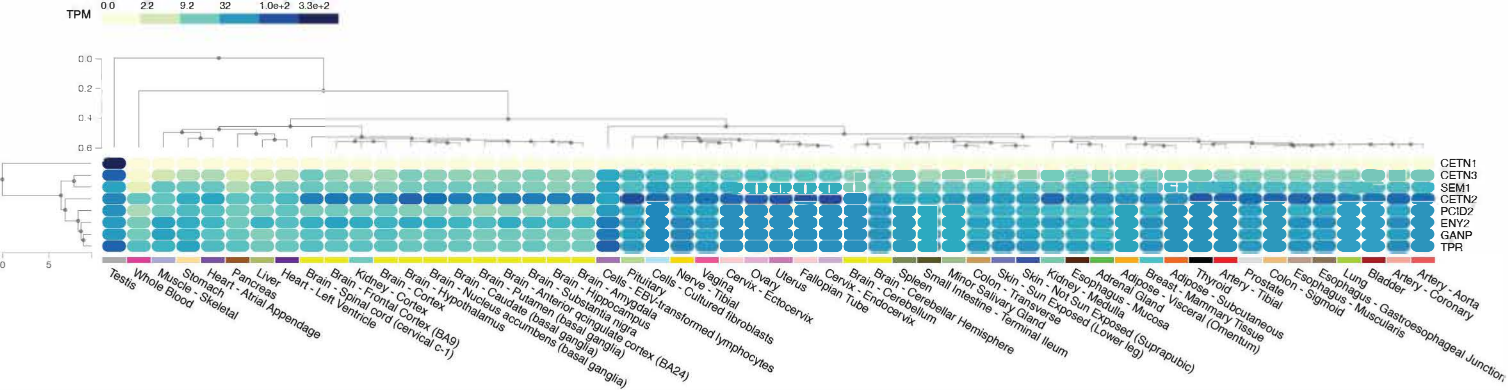

Supplementary Figure 1

a

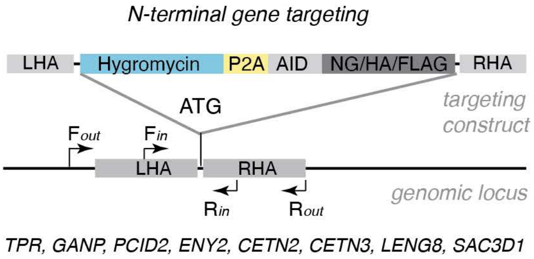

b

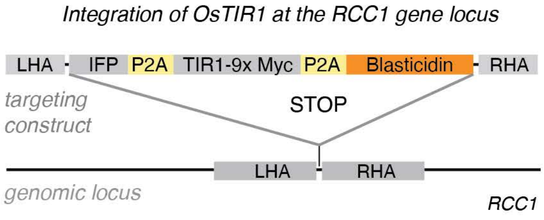

c

Gene	Homozygous AID-tagged cell line	Homozygous NG/mCherry-tagged cell line
TPR	AIDfl-NG, PMID: 32917881; TIR1 F74G, PMID: 33177522	PMID: 32917881
GANP	1xmicroAID-HA, PMID: 32917881;TIR1 positive	NG/mCherry_Nterm; TIR1 negative; Current study
PCID2	1xmicroAID-HA, PMID: 37085480;TIR1 positive	NG/mCherry_Nterm; TIR1 negative; Current study
ENY2	1xmicroAID-HA, Current study; TIR1 positive	NG/mCherry_Nterm; TIR1 negative; Current study
CETN2	1xmicroAID-HA, Current study; TIR1 positive	-
CETN2/3 Double	1xmicroAID-HA, Current study; TIR1 positive<br>1xmicroAID-FLAG, Current study; TIR1 positive	NG affects CETN3 protein localization
LENG8	1xmicroAID-HA; TIR1_F74G	NG_Nterm; TIR1 negative; Current study
SAC3D1	1xmicroAID-HA; TIR1_F74G	NG_Nterm; TIR1 negative; Current study

d

Protein	Endog. protein	AID-HA-tagged protein	NG/mCh.-tagged protein	Antibody
GANP	218.4 kDa	224.6 kDa	245.5 kDa	ab113295 (Abcam)
PCID2	46 kDa	52 kDa	73.1 kDa	ab216042 (Abcam)
ENY2	11.5 kDa	17.7 kDa	38.6 kDa	MA5-27843 (ThermoFisher)
CETN2	19.7 kDa	25.9 kDa	46.8 kDa	15877-1-AP (ProteinTech)
CETN3	19.5 kDa	25.7 kDa	46.6 kDa	15811-1-AP (ProteinTech)
LENG8	81.8 kDa	88 kDa	108.9 kDa	A304-947A (Bethyl)
SAC3D1	43.5 kDa	49.7 kDa	70.6 kDa	25857-1-AP (ProteinTech)

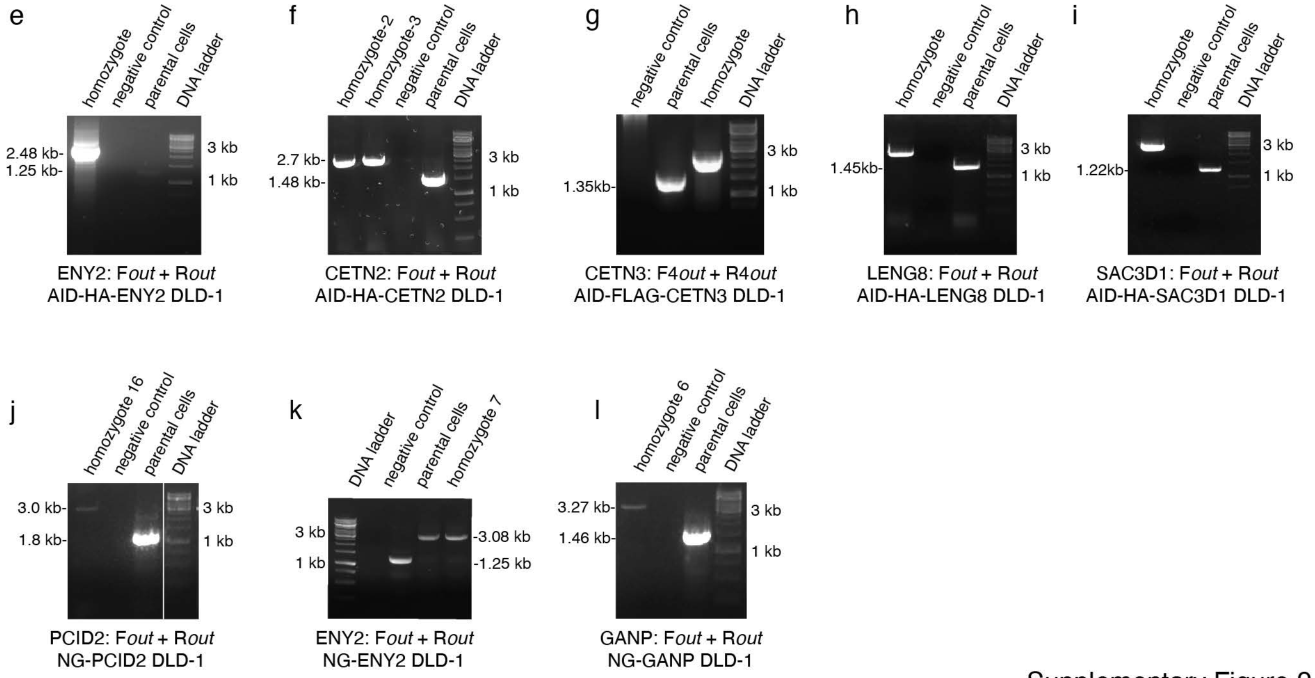

Supplementary Figure 2

bioRxiv preprint doi: https://doi.org/10.64898/2026.04.13.716336; this version posted April 14, 2026. The copyright holder for this preprint (which was not certified by peer review) is the author/funder. This article is a US Government work. It is not subject to copyright under 17 USC 105 and is also made available for use under a CC0 license.

a

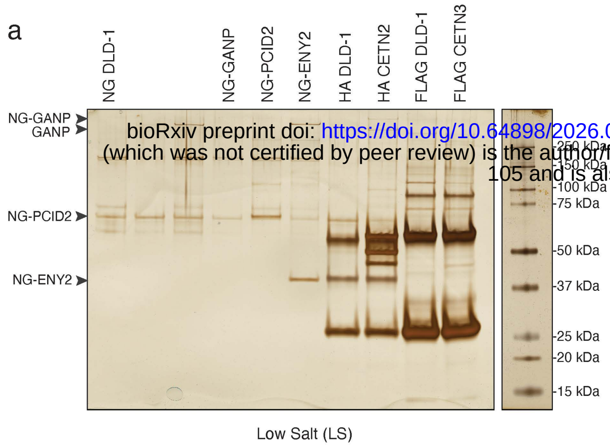

b

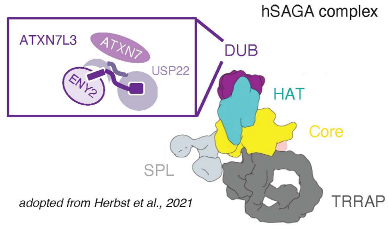

c

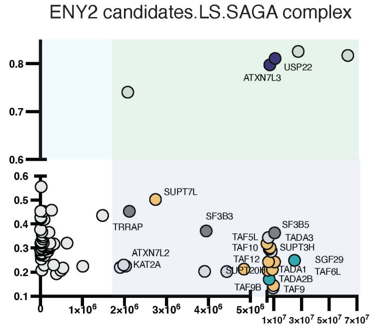

d

	PD score	Interacting surfaces (Chimera X)	PDB file
**SUPT20H**	**bait**		
TAF5L	0.8293558	Yes	7KTS
TRRAP	0.7951191	Yes	8H7G
TADA1	0.4012315	Yes, Small region	7KTS
ATXN7	0.4109231	Yes, Small region	7KTS
TAF9B	0.2175099	Yes, Small region	7KTS
TAF12	0.4111826	No	7KTS
SF3B3	0.3068855	No	7KTS
SUPT7L	0.2227782	No	8H7G
SF3B5	0.2836837	No	7KTS
SUPT3H	0.2153239	No	7KTS
TAF10	0.198901	No	7KTS
**TAF5L**	**bait**		
TAF6L	0.633167	Yes	7KTS
TAF9B	0.6086581	Yes	7KTS
SUPT7L	0.3185376	Yes, small region	8H7G, 7KTS
SF3B3	0.3851313	No	7KTS
**SUPT3H**	**bait**		
TADA1	0.7469953	Yes	7KTS
TAF10	0.7165584	Yes	7KTS
SUPT7L	0.7482181	Yes	8H7G
TAF12	0.6645459	Yes	7KTS
TAF9B	0.3428406	No	7KTS

e

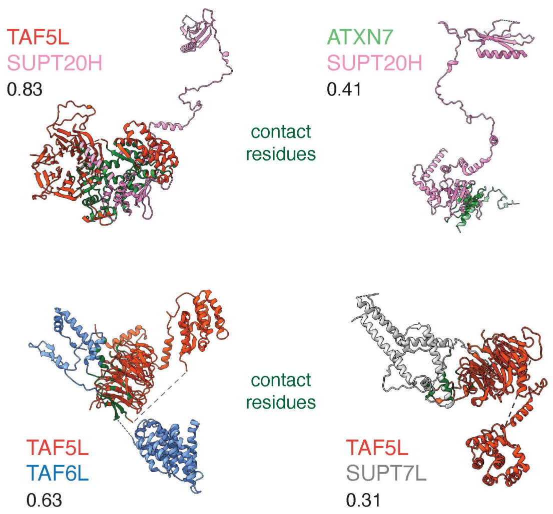

f g h

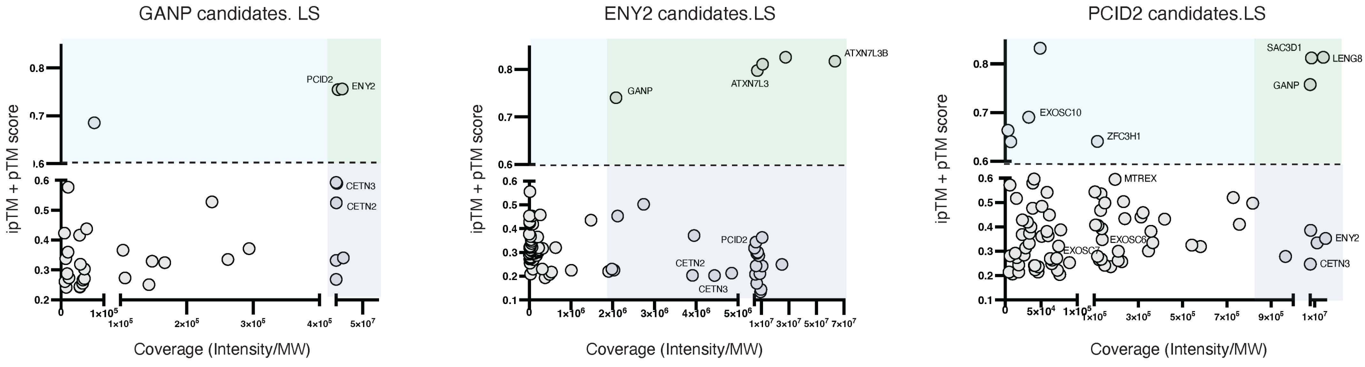

i

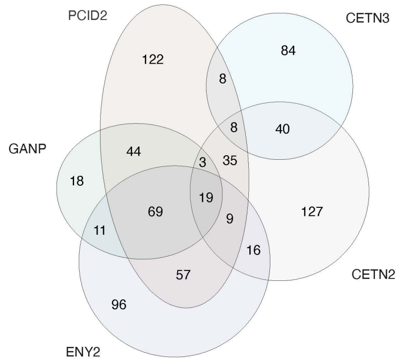

Supplementary Figure 3

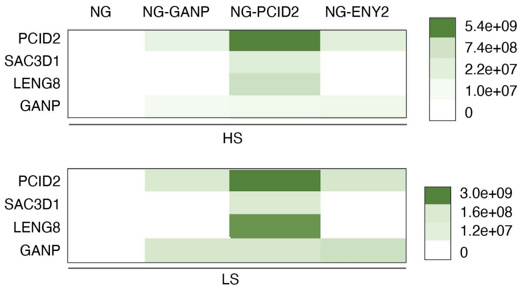

Supplementary Figure 4

bioRxiv preprint doi: https://doi.org/10.64898/2026.04.13.716336; this version posted April 14, 2026. The copyright holder for this preprint (which was not certified by peer review) is the author/funder. This article is a US Government work. It is not subject to copyright under 17 USC 105 and is also made available for use under a CC0 license.

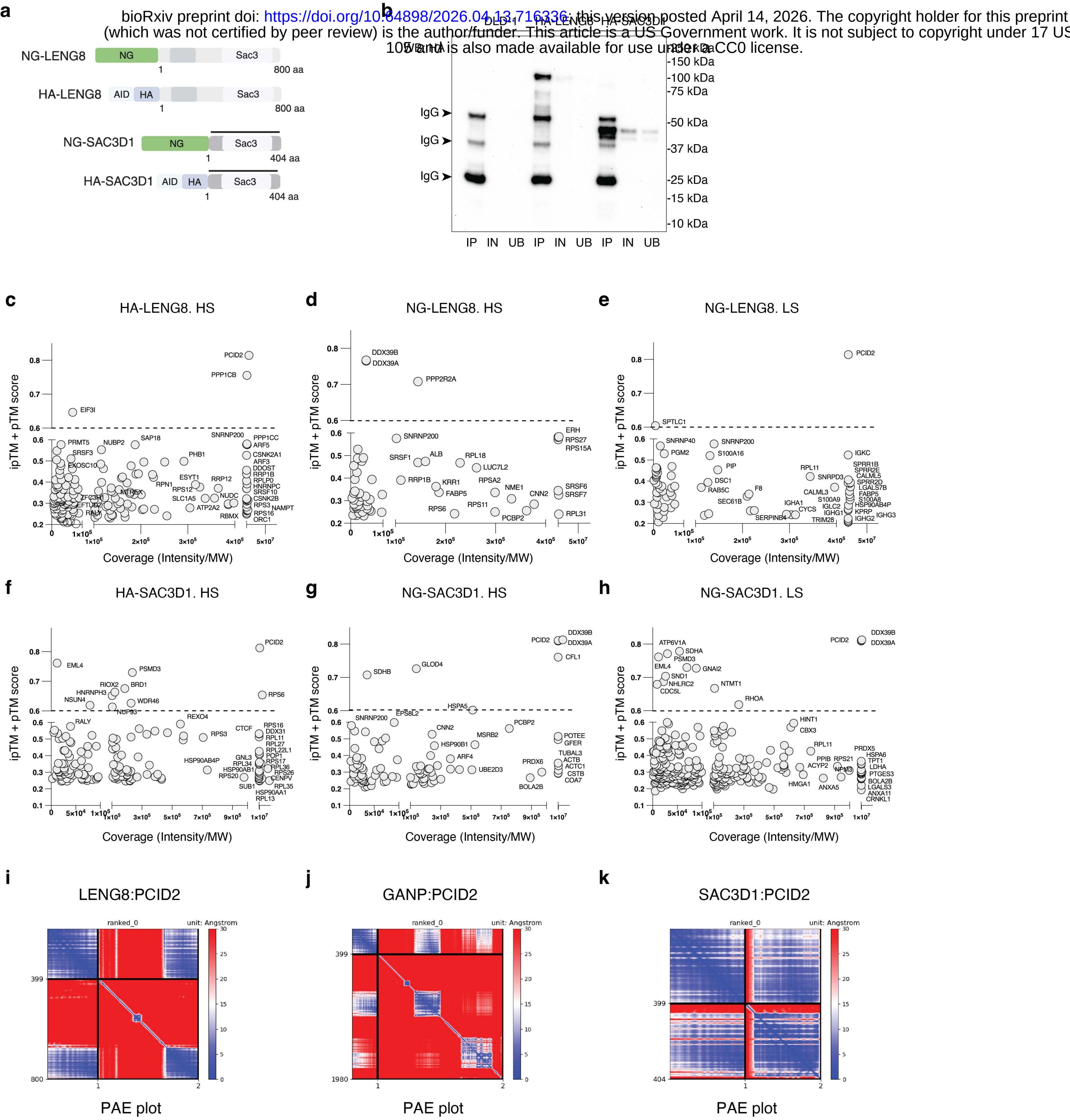

Supplementary Figure 5

bioRxiv preprint doi: https://doi.org/10.64898/2026.04.13.718336; this version posted April 14, 2026. The copyright holder for this preprint (which was not certified by peer review) is the author/funder. This article is a US Government work. It is not subject to copyright under 17 USC 105 and is also made available for use under a CC0 license.

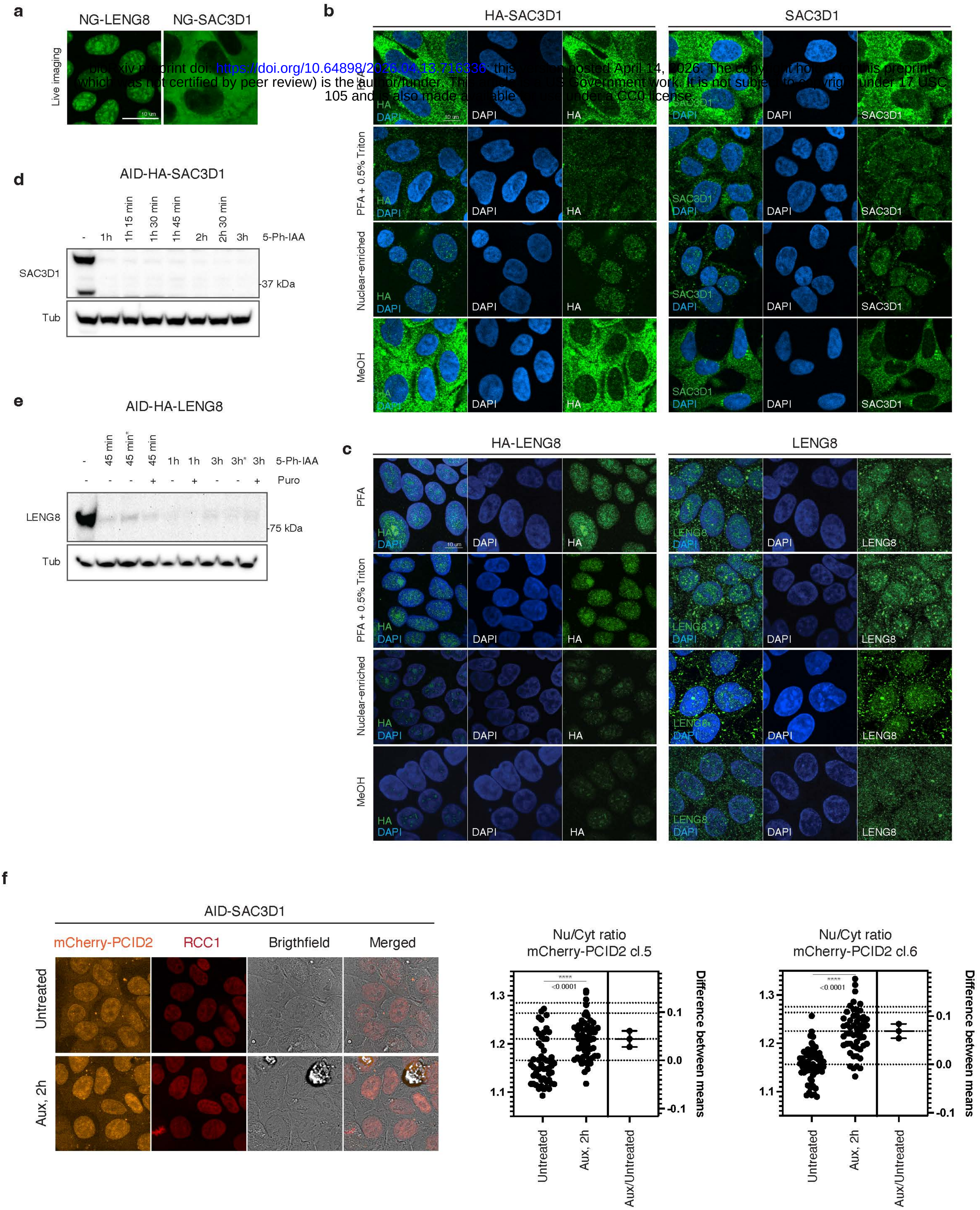

Supplementary Figure 6

bioRxiv preprint doi: https://doi.org/10.64898/2026.04.13.716336; this version posted April 14, 2026. The copyright holder for this preprint (which was not certified by peer review) is the author/funder. This article is a US Government work. It is not subject to copyright under 17 USC 105 and is also made available for use under a CC0 license.

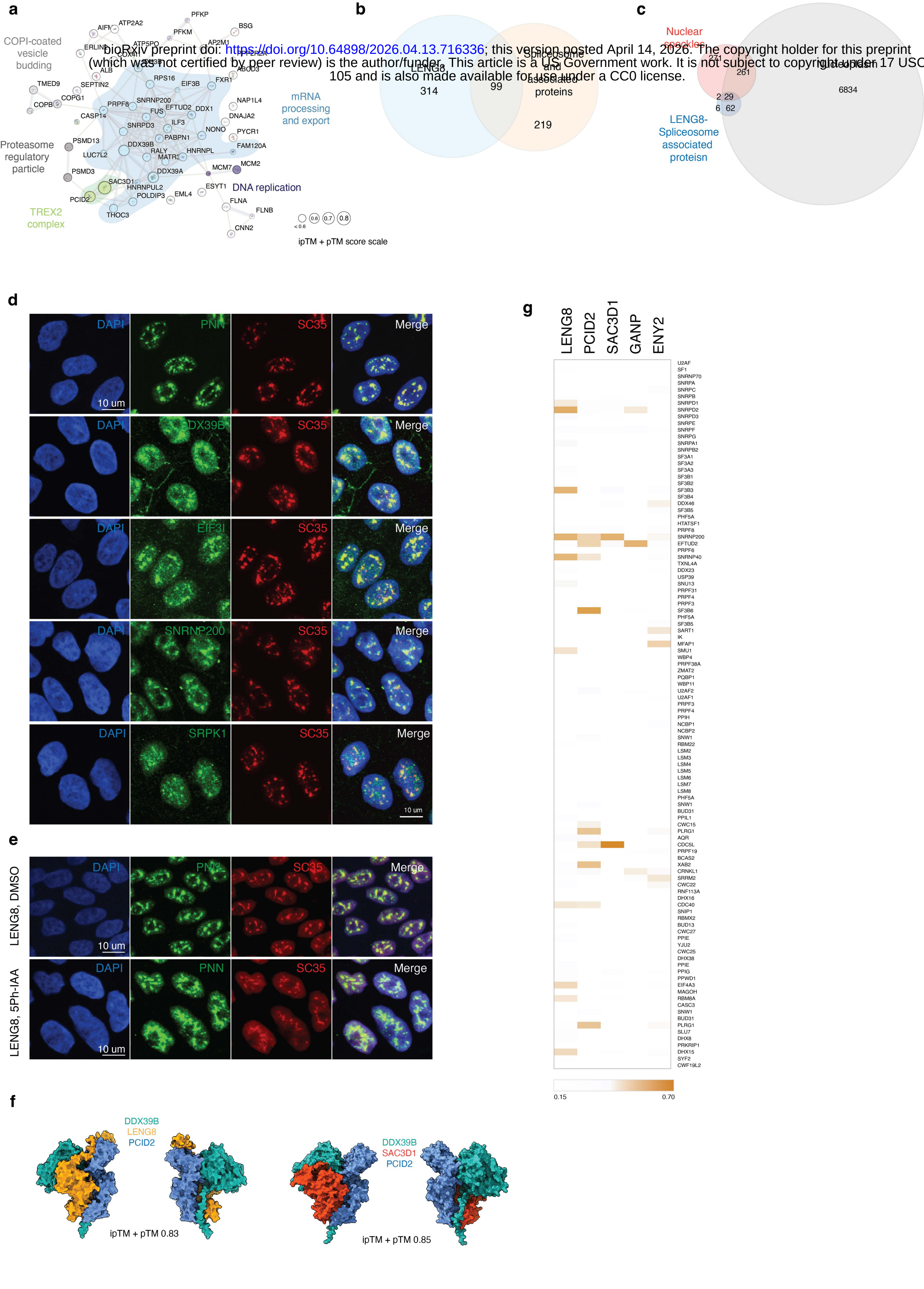

Supplementary Figure 7

a

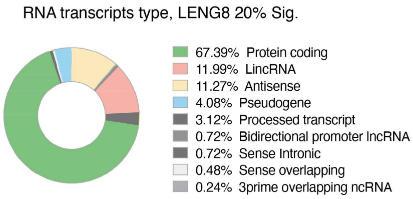

b

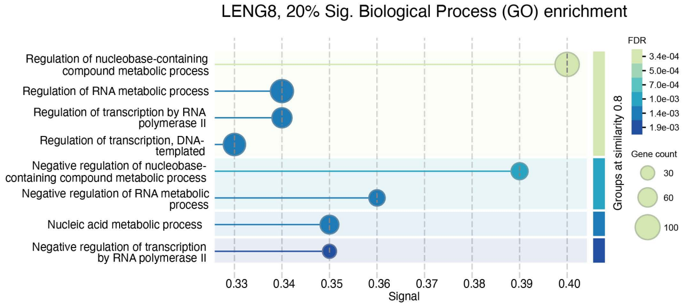

c

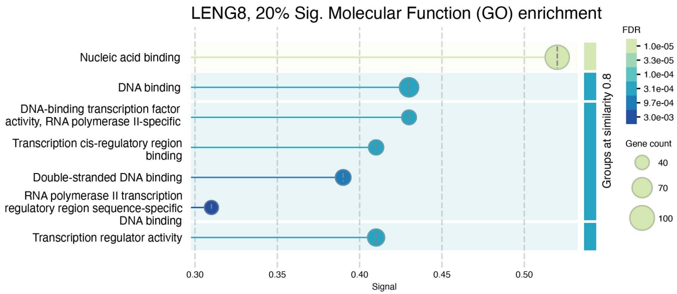

Supplementary Figure 8

bioRxiv preprint doi: https://doi.org/10.64898/2026.04.13.716336; this version posted April 14, 2026. The copyright holder for this preprint (which was not certified by peer review) is the author/funder. This article is a US Government work. It is not subject to copyright under 17 USC 105 and is also made available for use under a CC0 license.

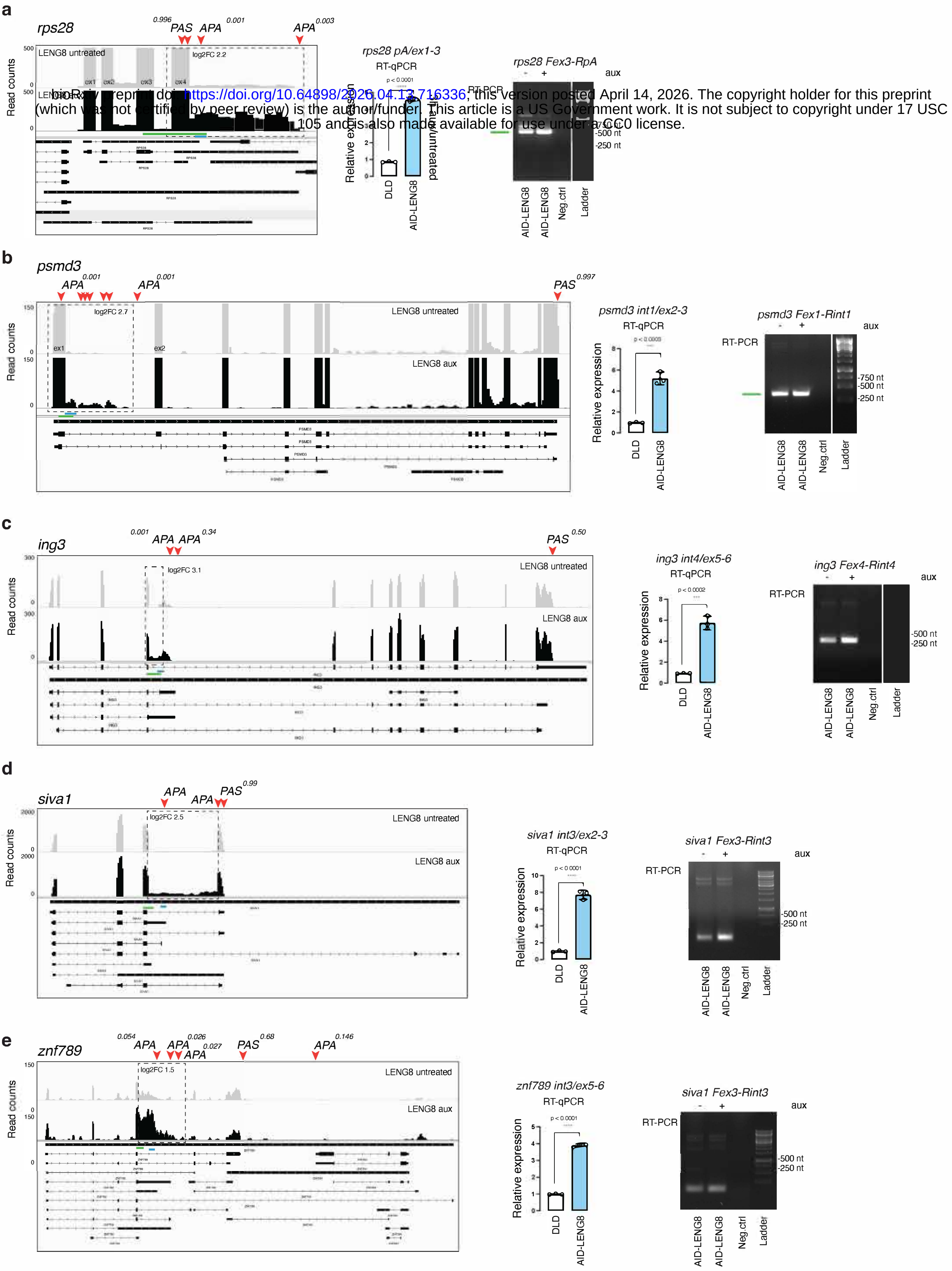

Supplementary Figure 9.
